# A *GNAS* Mutation Found in Pancreatic Intraductal Papillary Mucinous Neoplasms Induces Drastic Alterations of Gene Expression Profiles with Upregulation of Mucin Genes

**DOI:** 10.1371/journal.pone.0087875

**Published:** 2014-02-03

**Authors:** Hirotake Komatsu, Etsuko Tanji, Naoaki Sakata, Takeshi Aoki, Fuyuhiko Motoi, Takeshi Naitoh, Yu Katayose, Shinichi Egawa, Michiaki Unno, Toru Furukawa

**Affiliations:** 1 Institute for Integrated Medical Sciences, Tokyo Women's Medical University, Tokyo, Japan; 2 Department of Surgery, Tohoku University Graduate School of Medicine, Sendai, Japan; 3 Division of Integrated Surgery and Oncology, Tohoku University Graduate School of Medicine, Sendai, Japan; Columbia University, United States of America

## Abstract

*GNAS*, a gene encoding G protein stimulating α subunit, is frequently mutated in intraductal papillary mucinous neoplasms (IPMNs), which are indolent and slow-growing pancreatic tumors that secrete abundant mucin. The *GNAS* mutation is not observed in conventional ductal adenocarcinomas of the pancreas. To determine the functional significance of the *GNAS* mutation in pancreatic ductal lineage cells, we examined *in vitro* phenotypes of cells of pancreatic ductal lineage, HPDE, PK-8, PCI-35, and MIA PaCa-2, with exogenous expression of either wild-type or mutated (R201H) GNAS. We found that exogenous GNAS upregulated intracellular cyclic adenine monophosphate (cAMP), particularly in mutated *GNAS* transfectants, and upregulated expression of *MUC2* and *MUC5AC* in HPDE and PK-8 cells. By contrast, exogenous GNAS inhibited expression of mucin genes in PCI-35 and MIA PaCa-2 cells, despite upregulation of cAMP. We examined global gene expression profiles of some of the cells transfected with exogenous mutated *GNAS* (PK-8, PCI-35, and MIA PaCa-2), and found that PK-8 cells exhibited drastic alterations of the gene expression profile, which contrasted with modest alterations in PCI-35 and MIA PaCa-2 cells. To identify a cause of these different effects of exogenous mutated *GNAS* on phenotypes of the cells, we examined effects of interactions of the signaling pathways of G protein-coupled receptor (GPCR), mitogen-activated protein kinase (MAPK), and phosphatidylinositol 3-kinase (PI3K) on expression of mucin genes. The MAPK and PI3K pathways significantly influenced the expression of mucin genes. Exogenous GNAS did not promote cell growth but suppressed it in some of the cells. In conclusion, mutated *GNAS* found in IPMNs may extensively alter gene expression profiles, including expression of mucin genes, through the interaction with MAPK and PI3K pathways in pancreatic ductal cells; these changes may determine the characteristic phenotype of IPMN. PK-8 cells expressing exogenous mutated GNAS may be an ideal *in vitro* model of IPMN.

## Introduction

Intraductal papillary mucinous neoplasm (IPMN) of the pancreas is a cystic tumor consisting of dilated ducts lined by neoplastic cells secreting abundant mucin [1]. IPMN is regarded as a noninvasive precursor of ductal adenocarcinoma of the pancreas (PDAC). The prognosis of IPMN with an associated invasive carcinoma is poor, and it exhibits a 27–60% 5-year survival rate, depending on the extent and histological type of the invasive component [2]. Recently, somatic mutations in *GNAS* have been uncovered in IPMN, i.e., 41–66% of IPMNs harbor recurrent mutations in codon 201 of *GNAS*, mostly resulting in R201H or R201C in the protein [3], [4]. Furthermore, *GNAS* mutations are not found in conventional ductal adenocarcinomas or other cystic neoplasms of the pancreas [3], [4], [5]. Hence, mutated *GNAS* is considered a key molecule that distinguishes IPMN from other pancreatic tumors.

*GNAS* encodes guanine nucleotide-binding protein (G protein)-stimulating α subunit (G_sα_). G_sα_ forms a heterotrimeric G protein complex with the β and γ subunits and functions as a mediator in the G protein-coupled receptor (GPCR) signaling pathway. Binding of ligands to the receptor leads to G_sα_ activation, which involves an exchange of guanosine diphosphate (GDP) for guanosine triphosphate (GTP) and dissociation from the β and γ subunits. The activated G_sα_ transmits a stimulating signal to an effector, adenylyl cyclase, which produces cyclic adenosine monophosphate (cAMP). The latter binds to cAMP-dependent protein kinase (PKA), thereby activating PKA and the downstream signaling cascades [6]. G_sα_ has intrinsic hydrolytic activity that turns GTP to GDP, which inactivates G_sα_. The mutations of *GNAS* found in IPMN, R201H or R201C, are known to disrupt the intrinsic hydrolytic activity and result in constitutive activation of G_sα_ and its effector adenylyl cyclase, leading to autonomous synthesis of cAMP [7]. Somatic mutations in *GNAS* have been identified in various tumors besides IPMNs, including thyroid carcinomas, adrenocortical lesions, pituitary tumors, kidney tumors, Leydig cell tumors, intramuscular myxoma, and adenoma of the colorectum [7], [8], [9], [10], [11]. These organs have an endocrine or exocrine function, indicating that mutated GNAS is supposed to be associated with a secretory function. Nonetheless, the significance of GNAS in phenotypes of epithelial cells of the pancreatic duct requires elucidation. In this study, we examined the functional significance of mutated *GNAS* (found in IPMN) in cells of pancreatic ductal lineage *in vitro*.

## Materials and Methods

### Cell culture

The immortalized human pancreatic duct epithelial cell line, HPDE, established as described [12], was obtained from the original developer (Dr. M.-S. Tsao, Princess Margaret Hospital and Ontario Cancer Institute, Toronto, ON) and was cultured using Keratinocyte serum-free medium supplemented with bovine pituitary extract and epidermal growth factor (Life Technologies; Carlsbad, CA). The human pancreatic cancer cell lines, PK-8, PCI-35, and MIA PaCa-2, were obtained and cultured as follows: The PK-8 cell line, established as described [13], was obtained from the Cell Resource Center for Biomedical Research, Institute of Development, Aging and Cancer, Tohoku University, and was cultured using RPMI1640 with 10% fetal bovine serum (Sigma-Aldrich; St. Louis, MO). The PCI-35 cell line, established as described [14], was obtained from the original developer (Dr. Hiroshi Ishikura, Department of Pathology, Hokkaido University School of Medicine, Sapporo, Japan) and was cultured using RPMI1640 with 10% fetal bovine serum (Sigma). The MIA PaCa-2 cell line, established as described [15], was obtained from American Type Culture Collection (Manassas, VA) and was cultured using Dulbecco's modified Eagle medium with 10% fetal bovine serum (Sigma). All the cells were incubated in 5% CO_2_ at 37°C in an appropriate humid atmosphere. We confirmed mutations of exons 2 and 3 of *KRAS* and exons 8 and 9 of *GNAS* in these cells as described previously [8].

### Cloning and transfection of cDNA

We amplified cDNA of wild-type *GNAS* from a fetal brain cDNA library (Agilent Technologies Inc.; Santa Clara, CA) by means of polymerase chain reaction (PCR) using the following paired primers: C1, 5′-TTTAAGCTTCCGCCGCCGCCATGGGCTGC-3′ and C2, 5′-TTTCTCGAGGAGCAGCTCGTACTG-3′, and the KOD Plus DNA polymerase system (TOYOBO; Osaka, Japan). The amplified product was cloned into the pcDNA 3.1/V5-His expression vector (Life Technologies) at the *Hind*III and *Xho*I sites to generate the wild-type *GNAS-V5-His* vector. Site-directed mutagenesis to create *GNAS* (R201H)-*V5-His* was performed using M1, 5′-CCTGCTTCGCTGCC**A**TGTCCTGACTTCTGG-3′ and M2, 5′-AGAAGTCAGGACA**T**GGCAGCGAAGCAGGTC-3′ (bold letters indicate substitutions). The wild-type or mutant *GNAS*-V5-His cDNAs were also cloned into the pcDNA3.1/Hygro (+) expression vector (Life Technologies) at the *Hin*dIII and *Pme*I sites. The nucleotide sequences of the clones were verified using the BigDye terminator and Genetic Analyzer systems (Life Technologies). We carried out transfection of the vectors, pcDNA 3.1/V5-His-based vectors into PK-8, PCI-35, and MIA PaCa-2 cells and pcDNA 3.1/Hygro-based vectors into HPDE cells, using Lipofectamine 2000 reagent (Life Technologies) according to the manufacturer's recommendations. For assays using 6-well plates, 24 h before the transfection, PK-8, PCI-35, and MIA PaCa-2 cells were seeded at a density of 4×10^5^ cells/well and HPDE cells were seeded at 8×10^5^ cells/well. For assays using 96-well plates, the cells were seeded at 5×10^3^ cells/well 24 h before the transfection. After the transfection, the cells were incubated for 24 h and then collected and subjected to immunoblotting, cAMP analysis, and transcription assays including quantitative real-time PCR, serial analysis of gene expression (SAGE), semi-quantitative reverse transcription (RT)-PCR, and cell cycle analysis, as described below.

### Immunoblotting

The denatured total cell lysate was analyzed using electrophoresis on a 10–20% gradient polyacrylamide gel and blotted onto a polyvinylidene difluoride membrane (ATTO; Tokyo, Japan) using the XV Pantera MP System (DRC Co. Ltd.; Tokyo, Japan), according to the manufacturer's instructions. The primary antibodies used were a monoclonal anti-V5 (Life Technologies), monoclonal anti-G_sα_ (BD Biosciences; San Diego, CA), monoclonal anti-MAPK, activated (diphosphorylated ERK-1&2; Sigma-Aldrich), monoclonal anti-ERK2 (BD Biosciences), monoclonal anti-phospho-Akt (Cell Signaling Technology Inc.; Danvers, MA), monoclonal anti-Akt (Cell Signaling Technology Inc.), and a monoclonal anti-β-actin (Sigma-Aldrich). Blocking conditions and the concentrations of antibodies were determined according to the manufacturers' recommendations. The protein bands were visualized using the ECL Detection Reagent (GE Healthcare UK Ltd.; Buckinghamshire, UK) and captured digitally using an LAS 4000 Mini system (Fujifilm Co. Ltd.; Tokyo, Japan).

### Analysis of cAMP

Intracellular cAMP was measured using the cAMP EIA kit (Cayman Chemical Company; Ann Arbor, MI) according to the manufacturer's instructions. Measurements were normalized to total protein content of the samples. Each data point represented results of at least 3 independent experiments.

### Quantitative real-time PCR assay

Total RNA was isolated from cultured cells using the RNeasy Mini kit (Qiagen; Hilden, Germany). Complementary DNA was prepared by using High Capacity cDNA Reverse Transcription kit (Life Technologies) according to the manufacturer's instructions. The TaqMan Gene Expression Assay and the 7500 Real-Time PCR system (Life Technologies) were used to assess transcriptional expression according to the manufacturer's instructions. Plasmid vectors harboring the TaqMan PCR products were prepared using the StrataClone PCR Cloning kit (Agilent Technologies Inc.) according to the manufacturer's instructions and were used as standards for quantification in the real-time PCR assay. The expression of *MUC2* and *MUC5AC* was assessed relative to the endogenous expression of *GAPDH*. Each experiment included data from 3 independent wells of cells. The experiments were, at a minimum, independently duplicated.

### The colony formation assay

This assay of anchorage-dependent growth of cells was performed in PK-8, PCI-35, and MIA PaCa-2 cells as described previously [16]. The cells were transfected in 6-well plates, and then transferred onto 10-cm plates 24 h after the transfection. The selection agent G418 (Life Technologies) was added to the culture medium (400 µg/mL) 48 h after the transfection. Four weeks after the transfection, the cells were fixed with a 10% formalin solution and stained with hematoxylin. Colony area was assessed using the COLONY program (Fujifilm Co. Ltd.). Each experiment was performed using 3 dishes. The experiments were, at a minimum, independently duplicated.

### The cell proliferation assay

This colorimetric assay based on 3-[4,5-dimethylthiazol-2-yl]-2,5-diphenyltetrazolium bromide (MTT) was performed daily for 5 consecutive days (Days 0–4) using the Cell Proliferation Kit I (Roche Diagnostics; Basel, Switzerland) as described previously [17]. Cell proliferation was defined as the ratio of absorbance on Day 3 to that on Day 0. Each experiment included data from 8 independent transfection wells. The experiment was repeated, at a minimum, 2 times.

### The cell cycle assay

Cell cycle was assayed by measuring DNA content in cells stained with propidium iodide using the FACS Calibur System (BD Biosciences) as described previously [17]. The experiments were repeated, at a minimum, 2 times.

### Serial analysis of gene expression (SAGE)

This analysis was performed using a SOLiD SAGE kit and the massively parallel DNA sequencer 5500xl SOLiD system (Life Technologies) according to the manufacturer's instructions. XSQ files generated from the 5500xl SOLiD sequencer were converted into CSFASTA and QUAL files using XSQ Tools. Then, sequenced reads were aligned to the National Center for Biotechnology Information (NCBI) RefSeq reference sequence and SAGE tags were counted using SOLiD SAGE Analysis Software v1.10 (Life Technologies). The raw tag counts of individual genes were normalized by dividing them by the total tag counts, and were converted to obtain a value expressed in reads per million (RPM) tags [18], [19] using R software (http://www.r-project.org/). The values were calculated as binary logarithm values (log_2_ RPM). The SAGE data have been deposited in NCBI's Gene Expression Omnibus [20], [21] and are accessible through GEO Series accession number GSE53350 (http://www.ncbi.nlm.nih.gov/geo/query/acc.cgi?acc=GSE53350).

### Pathway analysis

Gene sets from the SAGE analysis were mapped onto the Pathway Map obtained from the Kyoto Encyclopedia of Genes and Genomes (KEGG) (http://www.genome.jp/kegg/) database [22].

### Semi-quantitative RT-PCR

Semi-quantitative PCR was performed using the Accuprime Taq polymerase system and the GeneAmp PCR system 9700 (Life Technologies) according to methods described previously [23]. Optimized cycling conditions were determined for each gene, and the expression of *GAPDH* served as an internal control. The primers used in the RT-PCR reactions are shown in Table S1. The intensity of bands was measured digitally using Image Gauge software (Fujifilm Co., Ltd.).

### U0126 and LY294002 treatment

U0126 (MERCK; Whitehouse Station, NJ), a potent inhibitor of mitogen-activated protein kinase kinase (MAP2K) [24], was dissolved in dimethyl sulfoxide (DMSO) and added to the culture medium (10 µM) 6 h after the transfection. The cells were harvested 48 h after the transfection and assayed. LY294002 (Cell Signaling), a specific inhibitor of phosphatidylinositol 3 (PI3) kinase [25], was dissolved in DMSO and added to the culture medium (50 µM) 24 h after the transfection. After 1-h incubation, the cells were harvested and assayed. In both experiments, DMSO was administered at the same concentration as a control. The experiment was repeated 2 times.

### Statistical analysis

Pearson's chi-squared test was used for the cell cycle analysis, and the Student's *t* test was used for data analysis of the other experiments. All calculations were performed using JMP 9 software (SAS Institute; Cary, NC). Statistical significance was assumed at p<0.05.

## Results

### Exogenous expression of GNAS in pancreatic ductal cells

**Figure 1:**
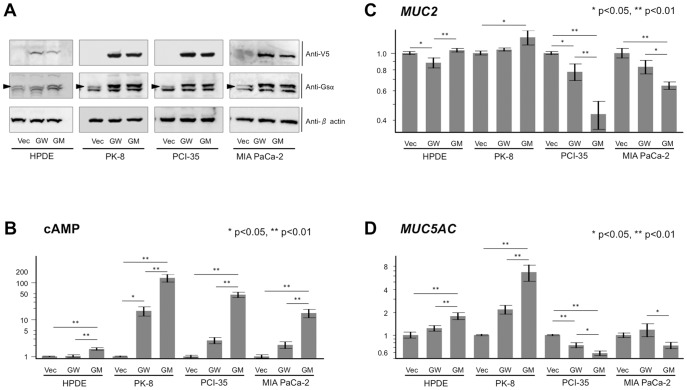
Exogenous GNAS increases cAMP levels and alters mucin gene expression in cells of pancreatic ductal lineage: the cell lines HPDE, PK-8, PCI-35, and MIA PaCa-2. (A) Immunoblots of total lysates of cells transfected with the empty vector (Vec), wild-type *GNAS-V5* (GW), or mutated *GNAS-V5* (R201H; abbreviated as GM) are shown on the right. Double bands were observed in assays with the anti-G protein α antibody, where the upper bands indicate specific immunoreactivity of G protein α (arrowheads). (B) Cyclic AMP levels were determined using an enzyme immunoassay. (C and D) Quantitative real-time PCR analysis of *MUC2* (C) and *MUC5AC* (D) expression. The data are shown on a logarithmic scale and values obtained are from independently duplicated experiments. Error bars indicate standard error. *p<0.05, **p<0.01.

First, we constructed expression vectors containing either wild-type or mutated (R201H) *GNAS* cDNA with a V5-tag sequence, and transfected them into the following cells of pancreatic ductal lineage: HPDE, PK-8, PCI-35, and MIA PaCa2. HPDE is an immortalized cell line derived from normal pancreatic ductal epithelial cells [12]. PK-8, PCI-35, and MIA PaCa2 are pancreatic cancer cell lines harboring the following gain-of-function mutations of *KRAS*: G12R in PK-8 cells, G12D in PCI-35 cells, and G12C in MIA PaCa-2 cells, but no mutations in the mutational hotspots of exons 8 and 9 (including codons 201 and 227) of *GNAS* [3]. We confirmed the exogenous expression of GNAS resulting from the transfection of expression plasmids by detecting the V5-tag and G_sα_ protein using immunoblotting (Fig. 1A). After transfection of the wild-type *GNAS* vector, the mutated *GNAS* vector, or an empty vector (as a control) into the cells, cAMP was quantified and compared. The transfection induced a significant increase in cAMP, especially in cells transfected with mutated *GNAS* (Fig. 1B). We also noted that the extent of the increase in cAMP production varied among the cell clones despite similar levels of expression of exogenous GNAS (except for HPDE cells). This result indicated that the transfected mutated *GNAS* functioned as expected but induced different levels of activation of cAMP signaling in these cell lines.

### The influence of exogenous GNAS on the expression of mucin genes

To determine the effects of mutated GNAS on the expression of mucin genes, we examined the expression of *MUC2* and *MUC5AC* (the genes encoding characteristic mucins abundantly expressed in IPMNs) in cells transfected with the empty vector, wild-type *GNAS*, or mutated *GNAS*, using real-time quantitative PCR. The results revealed that as a result of the transfection of mutated *GNAS*, the expression of *MUC2* and *MUC5AC* was significantly upregulated in HPDE and PK-8 cells, while it was downregulated in PCI-35 and MIA PaCa-2 cells (Fig. 1C and D). These results indicated that mutated GNAS changed the expression of mucin genes in a cell type-specific manner. We noted that the endogenous expression of *MUC2* and *MUC5AC* differed among the cell lines; *MUC5AC* was most abundantly expressed in PK-8 cells, suggesting that this cell line has an intrinsic activated *MUC5AC* expression pathway and is ready to respond to exogenous GNAS (Fig. S1).

### Proliferation of the cells expressing exogenous GNAS

**Figure 2:**
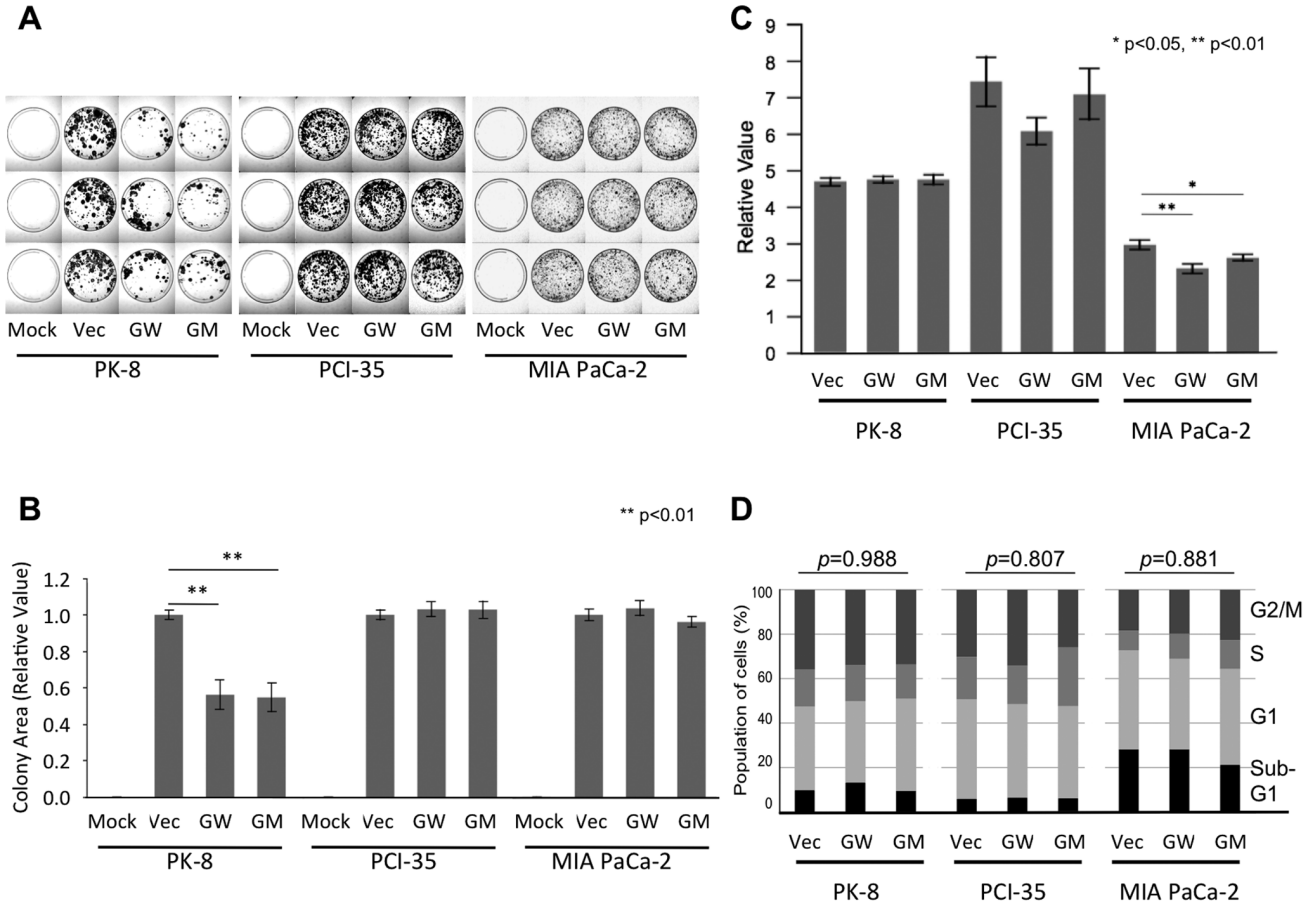
Exogenous GNAS does not stimulate cell proliferation or alterations of the cell cycle. Colony formation of cells transfected with the empty vector (Vec), wild-type *GNAS* (GW), or mutated *GNAS* (R201H; abbreviated as GM; panels A and B). (A) Images of cells incubated with a selective medium for 4 weeks. Mock denotes untransfected cells. (B) The mean values of total area of surviving colonies are shown as relative values compared to the control (Vec). Each transfection consisted of 3 plates, and mean data from independently triplicated (PK-8 cells) or duplicated (PCI-35 and MIA PaCa-2 cells) experiments were plotted with a range of one standard error. (C) A colorimetric proliferation assay showing cell proliferation after transient exogenous GNAS expression. Relative absorbance values (Day 3/Day 1) were plotted and are presented as mean ± SEM. (D) The cell cycle assay of cells with exogenous GNAS expression. *p<0.05, **p<0.01.

We performed a stable colony formation assay and a transient colorimetric proliferation assay to assess the effects of exogenous GNAS on *in vitro* anchorage-dependent cell proliferation. We found that exogenous GNAS, either wild-type or mutated, did not show a promotive effect on cellular proliferation and actually suppressed it in some cells (Fig. 2A–C). This result indicated that mutant *GNAS* did not produce an obvious advantage for cellular proliferation *in vitro*.

### Cell cycle characteristics of the cells with exogenous GNAS

We performed this assay to gain insight into the effects of exogenous GNAS on vital cellular processes. We did not observe significant alterations of the cell cycle (Fig. 2D).

### Global expression profiles under the influence of exogenous mutated GNAS

As demonstrated above, exogenous mutated GNAS had diverse effects on the expression of mucin genes in the examined cell lines, i.e., the effects appeared to be cell type-specific. To explore the details of these cell type-specific phenotypes caused by mutated GNAS, we performed global gene expression profiling by means of SAGE analysis using next-generation sequencing technology. We compared expression profiles between cells transfected with an empty vector (Vec) and cells transfected with mutated *GNAS* (GM) among the PK-8, PCI-35, and MIA PaCa-2 cell lines (GSE53350, NCBI's Gene Expression Omnibus database (http://www.ncbi.nlm.nih.gov/geo/)). In total, 15,841 genes were profiled, and they were gated to obtain markedly altered genes, i.e., those with expression ratios of GM/Vec either ≥4 or ≤0.25 (Fig. S2). We found that total numbers of the gated genes were different among the transfectant cells: 2258 in PK-8 cells, 260 in PCI-35 cells, and 66 in MIA PaCa-2 cells. These data indicated that PK-8 cells were sensitive, whereas PCI-35 and MIA PaCa-2 cells were not very sensitive to the effects of mutated GNAS in relation to alterations of gene expression. We validated the results of the SAGE analysis in PK-8 cells using semi-quantitative PCR (Fig. S3). The validation criteria are described in Fig. S3. Moreover, the data on genes previously described as highly upregulated or downregulated in IPMN and on genes known to be associated with mucin expression were also validated [26], [27], [28], [29].

### Expression profiles of mucin genes

In the SAGE data, we analyzed the effects of mutated GNAS on expression of mucin genes (Table S2). The human mucin gene family consists of members designated consecutively as *MUC1* through *MUC21*, and is classified into mucins of secreted or transmembrane forms [30]. Among the transfected cells studied, PK-8 cells exhibited the most consistent upregulation of mucin genes, including *MUC2*, *MUC5B*, *MUC6*, *MUC15*, and *MUC20*. Among these genes, *MUC2*, *MUC5B*, and *MUC6* encode secreted mucins and are known to be expressed abundantly in IPMNs [31], [32]. *MUC1*, which is commonly expressed in PDACs but relatively rarely in IPMNs [33], [34], was upregulated in PCI-35 and MIA PaCa-2 cells but was downregulated in the PK-8 cells. Thus, the PK-8 cells with exogenous mutated *GNAS* showed a contrasting pattern of expression between *MUC1* and *MUC2*, which is consistent with their mutually exclusive pattern observed in IPMN [35]. In the SAGE analysis, the upregulation of *MUC2* in PK-8 cells was observed as a result of the action of mutated GNAS, which was consistent with the results of the gene expression assays by means of real-time PCR as demonstrated in Fig. 1C. These results indicated that exogenous mutated GNAS globally altered expression of mucin genes; however, the direction and extent of the alterations were cell type-specific: PK-8 cells showed stronger upregulation of secreted mucin genes while PCI-35 and MIA PaCa-2 cells showed stronger upregulation of membranous mucin genes. These results suggested that PK-8 cells carrying mutated *GNAS* shared more phenotypic traits with IPMN than did the PCI-35- or MIA PaCa-2 transfectants. Information on *MUC5AC* expression was not available in the SAGE analysis because of the absence of a unique tag sequence for this analysis in our data-processing pipeline.

### Exogenous GNAS interacts with the PI3K-AKT and MAPK signaling pathways

**Figure 3:**
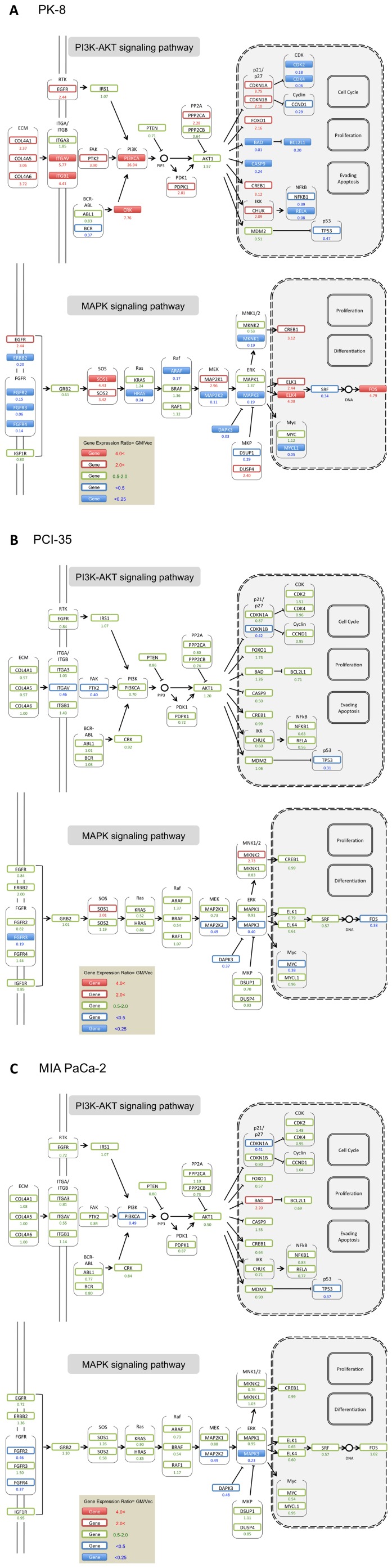
Alterations in gene expression related to signaling pathways as a result of exogenous mutated GNAS (R201H) expression in PK-8 (A), PCI-35 (B), and MIA PaCa-2 (C). Genes in the PI3K-AKT and MAPK signaling pathways in KEGG Mapper (http://www.genome.jp/kegg/) [22] were mapped with label colors according to the ratio of expression in cells carrying mutated *GNAS* (GM) to that in cells transfected with the empty vector (Vec).

To determine the significance of the alterations in gene expression patterns related to signaling pathways, the gated gene set (GM/Vec either ≥4 or ≤0.25) from the SAGE data was mapped onto the Pathways in Cancer in Pathway Mapping available from KEGG (http://www.genome.jp/kegg/) [22]. We found that the gated genes mainly belonged to the phosphoinositide 3-kinase (PI3K)-AKT signaling pathway and the mitogen-activated protein kinase (MAPK) signaling pathway, indicating that mutated GNAS induced alterations of expression of genes involved in these pathways (Fig. S4). We assessed the level of phosphorylated ERK and phosphorylated AKT in the cells transfected with the empty vector, wild-type *GNAS*, or mutated *GNAS*, and found that the extent of phosphorylation of these proteins was not changed significantly (Fig. S5). Next, we compared the detailed gene expression patterns in the MAPK and PI3K-AKT pathways among the cells in question (Fig. 3A–C). The results showed that the gene expression patterns pertaining to these signaling pathways were changed drastically in PK-8 cells but little in PCI-35 and MIA PaCa-2 cells as a result of action of exogenous mutated GNAS.

Although in PK-8 the expression of genes in these signaling pathways was altered considerably, the state of upstream and downstream genes from AKT or ERK was inconsistent. For instance, in the PI3K-AKT signaling pathway, genes encoding downstream molecules expressed in the nucleus were largely downregulated, whereas genes encoding upstream molecules were upregulated. By contrast, in the MAPK signaling pathway, genes encoding downstream molecules expressed in the nucleus were mainly upregulated, whereas genes encoding upstream molecules were mostly downregulated (Fig. 3A).

### Diverse mucin expression pathways and their relationship to GPCR, MAPK, and PI3K-AKT signaling

As demonstrated above, exogenous mutated GNAS induced upregulation of *MUC2* and *MUC5AC* in HPDE and PK-8 cells and downregulation of these genes in PCI-35 and MIA PaCa-2 cells. We also uncovered completely different patterns of gene expression related to the MAPK and PI3K-AKT pathways in response to mutated GNAS (which was supposed to activate the GPCR signaling pathway in all cell lines). Accordingly, we decided to elucidate the detailed interactions between these signaling pathways in terms of production of cAMP and control of the expression of *MUC2* and *MUC5AC*. To this end, we utilized PK-8 as GPCR-sensitive cells and PCI-35 as GPCR-less-sensitive cells: we transfected the cells with an empty vector, wild-type *GNAS*, or mutated *GNAS* and later administered either U0126, a potent MAP2K inhibitor [24], or LY294002, a specific inhibitor of PI3 kinase [25]. Next, we quantified cAMP and expression of *MUC2* and *MUC5AC*.

**Figure 4:**
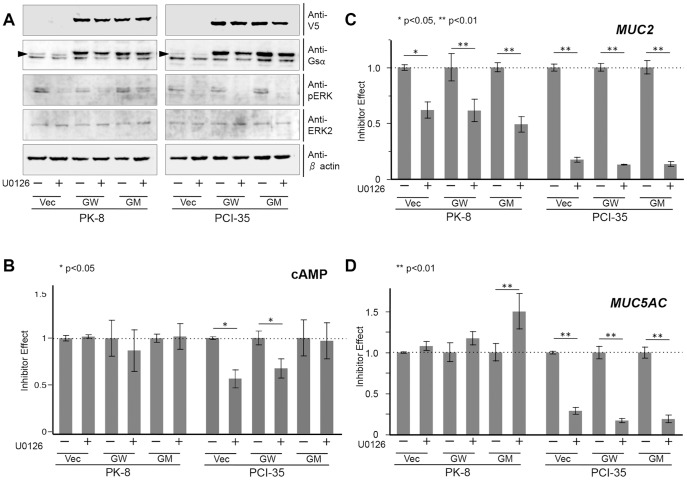
MAPK activity contributes to expression of mucin genes under different state of G protein activity. (A) Immunoblots of total lysates of cells transfected with the empty vector (Vec), wild-type *GNAS-V5* (GW), and mutated *GNAS-V5* (R201H; abbreviated as GM) with or without U0126, a potent mitogen-activated protein kinase kinase (MAP2K) inhibitor. (B) Cyclic AMP quantified using an enzyme immunoassay. U0126 treatment did not affect cAMP levels in PK-8 cells but downregulated cAMP in PCI-35 cells, except in the mutated *GNAS* transfectant. (C and D) A quantitative real-time PCR assay. (C) *MUC2* was consistently downregulated by U0126 in PK-8 and PCI-35 cells, regardless of the presence of exogenous *GNAS*. (D) *MUC5AC* was consistently downregulated in PCI-35 cells, regardless of the presence of exogenous *GNAS*, and upregulated by U0126 in PK-8 cells expressing exogenous mutated GNAS. Values obtained from independently duplicated experiments were plotted. Error bars indicate standard error. *p<0.05; **p<0.01.

The U0126 treatment inhibited phosphorylation of ERK in PK-8 and PCI-35 cells, regardless of the type of transfected plasmid (Fig. 4A). U0126 did not cause significant alterations in cAMP in the PK-8 cells with any of transfection but downregulated cAMP in PCI-35 cells transfected with either the empty vector or wild-type *GNAS* (Fig. 4B). As for the expression of mucin genes, the U0126 treatment consistently downregulated *MUC2* expression in both PK-8 and PCI-35 cells; however, there were inconsistent effects on the expression of *MUC5AC*, i.e., upregulation in PK-8 cells carrying mutated *GNAS* but downregulation in all PCI-35 transfected cells (Fig. 4C and D). This result indicated that phosphorylated ERK (p-ERK) played little or no role in cAMP production, a stimulatory role in *MUC2* expression, and a suppressive role in *MUC5AC* expression in PK-8 cells; at the same time, p-ERK stimulated cAMP production and the expression of *MUC2* and *MUC5A*C in PCI-35 cells.

**Figure 5:**
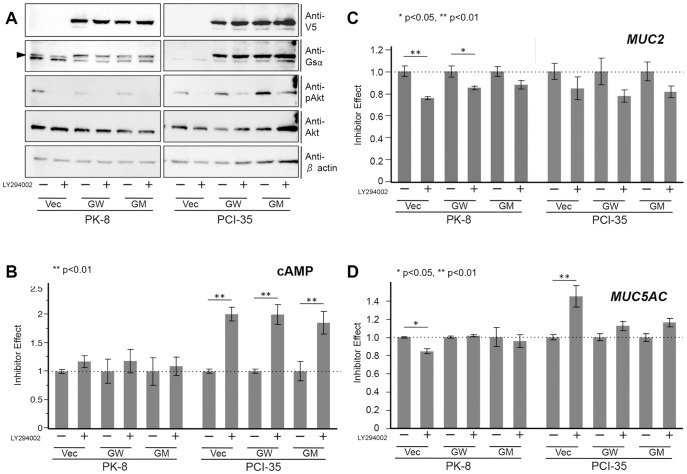
PI3K-AKT activity influences mucin gene expression under different state of G protein activity. (A) Immunoblots of total lysates of cells transfected with the empty vector (Vec), wild-type *GNAS-V5* (GW), and mutated *GNAS-V5* (R201H; abbreviated as GM) with or without LY294002, a specific inhibitor of PI3 kinase. (B) Cyclic AMP measured by means of an enzyme immunoassay. The cAMP production was not significantly affected by LY294002 in PK-8 cells but was upregulated in PCI-35 cells. (C and D) A quantitative real-time PCR assay. *MUC2* is modestly downregulated by LY294002. The latter downregulated *MUC5AC* in PK-8 cells but upregulated it in PCI-35 cells; the effect was not significant in the PCI-35 clone expressing exogenous GNAS. Values obtained from independently duplicated experiments were plotted. Error bars indicate standard error. *p<0.05, **p<0.01.

The LY294002 treatment inhibited phosphorylation of AKT in PK-8 and PCI-35 cells, regardless of the type of transfected plasmid (Fig. 5A). LY294002 upregulated cAMP modestly in PK-8 but markedly in PCI-35 cells (Fig. 5B). As for the expression of mucin genes, the LY294002 treatment consistently downregulated *MUC2* expression in both cell lines; significantly in PK-8 cells transfected with either the empty vector or wild-type *GNAS* but not significantly in the PK-8 carrying mutated *GNAS* and in all PCI-35 transfectant cells. LY294002 had inconsistent effects on *MUC5AC* expression: downregulation in PK-8 cells but upregulation in PCI-35 cells, the latter effect being significant only in the cells transfected with the empty vector (no exogenous *GNAS*; Fig. 5C and D). These results indicated that in PK-8 cells, phosphorylated AKT was not involved in cAMP production but stimulated *MUC2* and *MUC5AC* expression. At the same time, in PCI-35 cells, p-AKT strongly suppressed cAMP production, modestly increased *MUC2* expression, and inhibited *MUC5AC* expression (the latter effect was significant in the absence of exogenous *GNAS*).

## Discussion

We examined *in vitro* phenotypes of cell lines of pancreatic ductal lineage, HPDE, PK-8, PCI-35, and MIA PaCa-2, with exogenous expression of either wild-type or mutated GNAS (R201H). We found that exogenous GNAS upregulated cAMP, particularly in mutated *GNAS* transfectants, and upregulated expression of *MUC2* and *MUC5AC* in HPDE and PK-8 cells. On the other hand, the exogenous GNAS downregulated expression of the mucin genes in PCI-35 and MIA PaCa-2 cells, despite upregulation of cAMP. We subsequently examined global gene expression profiles of PK-8, PCI-35, and MIA PaCa-2 cells after transfection of mutated *GNAS* and found that PK-8 cells showed a drastic alteration of the gene expression profile by exogenous mutated GNAS, which contrasted with the modest alterations observed in PCI-35 and MIA PaCa-2 cells.

To identify a cause of these different effects of exogenous mutated GNAS on phenotypes of the cell lines, we examined effects of interactions of the GPCR, MAPK, and PI3K signaling pathways on expression of mucin genes. The results showed that the MAPK and PI3K pathways significantly influenced the expression of mucin genes. Furthermore, we found that exogenous GNAS did not promote cell growth but actually suppressed it in some of the cell lines.

The R201H mutation of GNAS is highly specific for IPMN among pancreatic tumors, and the most characteristic feature of IPMN is excessive production of mucin. Accordingly, we hypothesized that mutated GNAS would enhance mucin gene expression in pancreatic ductal cells. To characterize phenotypic changes caused by the mutated GNAS in pancreatic ductal cells, we employed HPDE cells (an immortalized cell line derived from healthy pancreatic duct epithelial cells) and pancreatic cancer cell lines (PK-8, PCI-35, and MIA PaCa-2) carrying *KRAS* mutations. HPDE was expected to show the “pure” phenotype of mutated *GNAS*, whereas the pancreatic cancer cells were expected to manifest the phenotype of mutated *GNAS* plus mutated *KRAS* (the latter corresponds to common mutations found in IPMN) [3], [4]. We demonstrated that cAMP was upregulated by exogenous GNAS, particularly by mutated GNAS; however, the degree of elevation varied considerably among the cell lines. Farther downstream, the exogenous GNAS induced alterations of mucin gene expression, strongly in PK-8 cells and modestly in HPDE, PCI-35, and MIAPaCa-2 cells. PK-8 cells showed strong intrinsic expression of *MUC5AC* and consistent upregulation of mucin genes by the exogenous GNAS along with the upregulation of cAMP. HPDE cell clones also showed consistent but weaker upregulation of cAMP and of mucin genes compared to PK-8 cells. The reduced degree of response may be due to the lower level of expression of exogenous *GNAS* in HPDE cells. By contrast, PCI-35 and MIAPaCa-2 cells showed inconsistent responses to exogenous GNAS: elevation of the cAMP level but downregulation of mucins. These diverse reactions to exogenous GNAS among these cell lines indicate that activation of G protein signaling may induce consistent upregulation of cAMP production but diverse effects downstream of cAMP, which may result in different functional consequences of mutated GNAS in pancreatic ductal cells. Mutated GNAS induced alterations of global gene expression profiles, drastically in PK-8 cells but modestly in PCI-35 and MIA PaCa-2 cells. PK-8 with exogenous GNAS showed more resemblance to the expression of mucin genes in IPMN than did PCI-35 and MIA PaCa-2 cells with exogenous GNAS. These results suggest that PK-8 cells may have a robust and somewhat active intrinsic G protein signaling system, and that these cells are ready to respond to exogenous GNAS more vigorously than are either PCI-35 or MIAPaCa-2 cells. In this regard, mutated *GNAS* found in IPMN is expected to promote abundant mucin secretion under active control of a robust GPCR signaling pathway. Thus, PK-8 cells expressing mutated GNAS seem to share phenotypic traits with IPMN, and therefore, PK-8 with exogenous mutated GNAS is likely to be an ideal *in vitro* model of IPMN.

**Figure 6:**
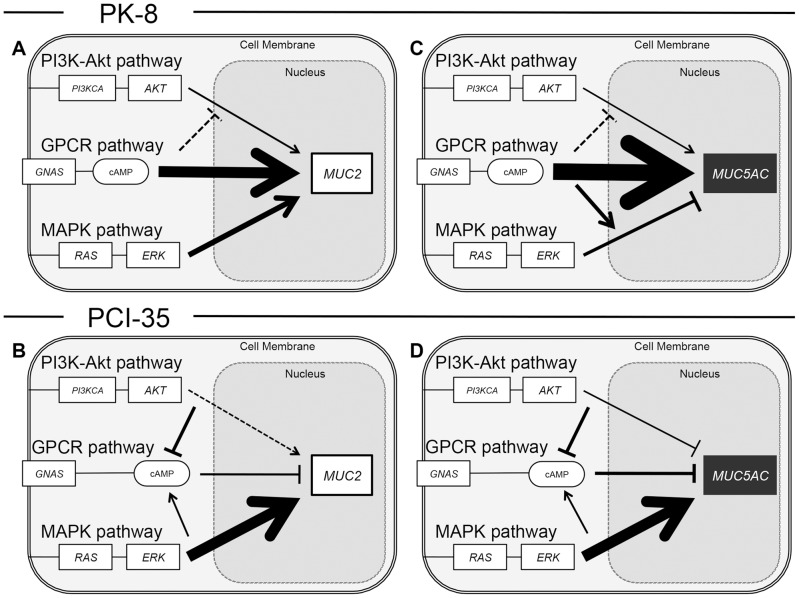
Diverse effects of signaling pathways on *MUC2* and *MUC5AC* expression in PK-8 and PCI-35 cells. (A) The regulation of *MUC2* expression in PK-8 cells was predominantly mediated by the GPCR pathway, with synergistic effects of the MAPK pathway and additive effects of the PI3K pathway. Minimal interactions existed among the 3 signaling pathways themselves. (B) *MUC2* expression in PCI-35 cells was regulated predominantly by the MAPK pathway and additively by the PI3K pathway, whereas the GPCR pathway was antagonistic. Active interactions existed among the signaling pathways: cAMP was upregulated by active MAPK and downregulated by active PI3K. (C) *MUC5AC* expression in PK-8 cells was regulated predominantly by the GPCR pathway, the MAPK pathway was antagonistic, and the PI3K pathway played a weak role. (D) *MUC5AC* expression in PCI-35 cells was regulated predominantly by the MAPK pathway, whereas both the PI3K and GPCR pathways were antagonistic.

We aimed to determine how the GPCR, MAPK, and PI3K-AKT signaling pathways interact with respect to the regulation of mucin gene expression. In PK-8 cells, mutated GNAS induced alterations of expression of genes involved in the MAPK and PI3K-AKT signaling pathways, as shown in Figure 3. Although ERK and AKT were unaltered, genes in their upstream and downstream pathways were altered drastically. This result is consistent with our immunoblot assay showing that the level of phosphorylation of ERK and AKT was unchanged. Although mechanistic insight into this phenomenon is outside the scope of this study, these results suggest that upregulation of the GPCR pathway could alter expression of genes involved in the MAPK and PI3K-AKT signaling pathways without obvious changes in either ERK or AKT. Chemical inhibition of phosphorylation of ERK and AKT in our experiments shed some light on the interactions between mucin genes and these signaling pathways. The results shown in Fig. 4 indicate that *MUC2* expression was upregulated by phosphorylated ERK in PK-8 and PCI-35 cells regardless of *GNAS* status or the cAMP level. These data pointed to a consistent synergistic effect of MAPK activity with G protein signaling on *MUC2* expression (Fig. 6A and B).

On the other hand, *MUC5AC* expression was interpreted as downregulated by phosphorylated ERK in PK-8 cells, particularly in the cells with mutated GNAS, but *MUC5AC* expression seemed to be upregulated in PCI-35 cells regardless of *GNAS* status or the cAMP level. These data suggested that active MAPK may interfere with hyperactive G protein signaling in PK-8 cells, whereas in PCI-35 cells, MAPK may have synergistic effects with G protein signaling on *MUC5AC* expression (Fig. 6C and D). The results displayed in Fig. 5 regarding inhibition of phosphorylation of AKT indicated that *MUC2* expression was upregulated by active PI3K-AKT signaling in PK-8 and PCI-35 cells; however, exogenous GNAS appeared to attenuate this effect in PK-8 cells. These results indicated that regulation of *MUC2* expression by G protein signaling and PI3K-AKT signaling was additive in PK-8 cells and slightly synergistic in PCI-35 cells (Fig. 6A and B). On the other hand, *MUC5AC* expression was upregulated in PK-8 cells but downregulated in PCI-35 cells by active PI3K-AKT without exogenous GNAS, whereas those effects seemed to be attenuated by exogenous GNAS in both cell lines. This observation indicated that there was some antagonism between PI3K-AKT signaling and G protein signaling in these cells in relation to *MUC5AC* expression (Fig. 6C and D). These results show predominant GPCR-dependency of mucin gene expression in PK-8 cells, and this feature may resemble the phenotype of IPMN. Hence, upregulation of *MUC2* and *MUC5AC* by mutated GNAS in PK-8 cells may provide important clues to the fundamental pathobiological features of IPMN. By contrast, PCI-35 and MIAPaCa-2 cells appear to be less dependent on the GPCR pathway but more dependent on the MAPK and PI3K-AKT pathways in the expression of mucins, and this trait may resemble the phenotype of PDAC.

The exogenous mutated *GNAS* did not promote *in vitro* cell proliferation. This finding indicates that mutated GNAS alone may not be sufficient to induce or maintain infinite growth, and this observation is consistent with the finding that the genetically engineered mouse model of mutated *GNAS* does not develop tumors without synergistic molecular aberrations [36]. Instead, exogenous *GNAS* inhibited proliferation of some cell clones. This phenomenon may be related with the indolent nature of IPMN compared to PDAC, the latter being usually free of *GNAS* mutations [37].

Some genes with altered expression patterns induced by exogenous mutated *GNAS* are of interest for understanding the phenotypes associated with the upregulation of GPCR signaling. *ALDHA1* encodes aldehyde dehydrogenase 1 family member A1, which serves as a marker of cancer-initiating cells and correlates with tumorigenicity [38]. *CD55* or *DAF*, the decay-accelerating factor, encodes a protein of the membrane-bound complement regulatory protein family, which is a known target of the MAPK pathway [39], and its presence in this dataset is consistent with the fact that most of the genes located downstream of the MAPK pathway were upregulated. *CREB1* encodes cAMP responsive element-binding protein 1 (CREB1), which functions as a transcription factor that is primarily controlled by PKA but also by MSK-1 in the MAPK pathway, and by AKT in the insulin-like growth factor pathway [40], [41], [42]. CREB1 can modify the expression of diverse proteins involved in metabolism, transcription, and the cell cycle, as well as expression of neuropeptides, growth factors, and mucins [27], [28], [29]. *DDIT4* encodes DNA damage-inducible transcript 4, also known as *REDD1*, which is a negative regulator of mammalian target of rapamycin (mTOR) and a possible tumor suppressor in renal cell carcinoma [43], [44]. The increase in *DDIT4* transcription observed in PK-8 cells as a result of the action of mutated *GNAS* may be indicative of a suppressive effect of *GNAS* on cell proliferation. *GNB2* encodes G protein β polypeptide 2, which is associated with the NF-κB signaling pathway and tumorigenesis [45]. *GNG10* encodes G protein subunit γ 10 and is mutated in melanoma [46]. *LCN2*, also known as *NGAL*, encodes lipocalin 2, which is a neutrophil gelatinase-associated lipocalin that contributes to tumor progression [47]. Lipocalin 2 is also known as an adipokine that is upregulated by insulin via the PI3K and MAPK signaling pathways [48]. *MIA3*, also known as *TANGO*, encodes melanoma inhibitory activity family member 3, which exhibits tumor suppressor properties in colon and hepatocellular carcinomas and in malignant melanoma [49]. *PHLPP1* encodes PH domain and leucine-rich repeat protein phosphatase 1, which dephosphorylates AKT [50] and suppresses the growth of colon cancer and glioblastoma cells [51]. *PIK3CA* encodes phosphatidylinositol-4,5-bisphosphate 3-kinase catalytic subunit α, which is mutated in many types of human tumors, including IPMNs [52], [53].

As we demonstrated in this work, mutant GNAS can enhance the expression of mucin genes in cells of pancreatic ductal lineage. Excessive production of mucin is a hallmark of IPMN and is associated with severity of symptoms in patients with this type of tumor. Mucin causes acute or chronic obstructive pancreatitis by clogging up the pancreatic duct and hampering pancreatic juice drainage. The obstructive pancreatitis may eventually progress to pancreatic dysfunction with exocrine and endocrine insufficiency. Treatments targeting the active G protein signaling driven by mutated GNAS may decrease the excessive secretion of mucin, relieve the symptoms, and impede progression to pancreatic dysfunction.

IPMNs are classified into 4 distinct pathological types based on histomorphological variations of neoplastic papillae and on the expression of distinct mucin proteins: gastric, intestinal, pancreatobiliary, and oncocytic [31]. MUC5AC is a common type of mucin for any type of IPMN, whereas MUC2 is specific to intestinal-type IPMN. The intestinal-type IPMN is associated with mucinous colloid carcinoma, which has a better prognosis than does tubular adenocarcinoma, which is usually associated with gastric- or pancreatobiliary-type IPMN. Hence, expression of distinct mucin proteins is an important prognostic marker of IPMN. In our study, *MUC2* and *MUC5AC* appeared to be distinctively regulated by either synergistic or antagonistic interactions of the GPCR, PI3K-AKT, and MAPK signaling pathways. These results suggest that the interaction of signaling pathways may underlie not only the regulation of mucin expression, but also progression of IPMNs to different types of carcinoma (some of them more invasive than others). Further studies of mucin expression pathways focusing on promoter function may elucidate the distinct processes of mucin expression in IPMN and PDAC; this new knowledge may provide insight into the mechanisms underlying phenotypes of pancreatic neoplasms and carcinomas.

In conclusion, mutated GNAS found in IPMNs may extensively alter gene expression profiles, including expression of mucin genes, as a result of an overactive GPCR signaling pathway and its interactions with the MAPK and PI3K pathways in pancreatic ductal cells; these changes may determine the characteristic phenotype of IPMN.

## Supporting Information

Figure S1 ***MUC2*** **and** ***MUC5AC*** **expressions among vector-transfectants.** *MUC2* and *MUC5AC* gene expression were evaluated by the quantitative real-time PCR method. Absolute values of each gene expression were converted into relative values to a value of *MUC2* expression of HPDE vector-transfectant. Values of independently duplicated experiments were plotted with the range of one standard error and statistically compared. Two asterisks indicate p<0.01.
(TIF)

Figure S2 **An algorithm for data processing in SAGE analysis.**
(TIF)

Figure S3 **(A) Validation of SAGE data by semiquantitative PCR assay.** Total RNA obtained from PK-8 transfected with vector (Vec) or the mutated *GNAS* (G201H) (GM), which was used in SAGE analysis, was reverse-transcribed and used for PCR with primers listed in Table S1. The optimized cycling conditions were determined for each gene, and the expression of *GAPDH* was represented as an internal control. Intensities of bands were digitally measured and illustrated with bar graphs below the images. Intensity of GM was demonstrated as relative value to Vec (%). Gene expression values in the SAGE analysis were shown at the bottom. (B) Selection criteria for validation. Specially considering the genes associated with mucin gene expressions, genes showing inverse expression between PK-8 and other cell lines (PCI-35 and MIA PaCa-2 cells) were nominated, because they demonstrated completely converse *MUC2* and *MUC5AC* reactions to exogenously expressed *GNAS* in real-time qPCR experiments. Genes previously described as being highly upregulated or downregulated in IPMN and genes known to be associated with the expression of mucin were also validated.
(TIF)

Figure S4 **Alterations of gene expressions in signaling pathways.** Genes of altered expressions in the ratio of the mutated *GNAS* transfectants to vector transfectants (GM/Vec) ≥4 or ≤0.25 in PK-8 in SAGE data were mapped on “Pathways in Cancer” in “Pathway Mapping” obtained from KEGG (http://www.genome.jp/kegg/). Panel A indicates upregulated genes while panel B indicates downregulated genes.
(TIF)

Figure S5 **Immunoblots of total lysates of cells transfected with the vector (Vec), the wild-type** ***GNAS*** **-V5 (GW), and the mutated** ***GNAS*** **-V5 (R201H) (GM) probed with antibodies indicated in the right column.**
(TIF)

Table S1 **Primers used for RT-PCR reactions.**
(DOCX)

Table S2 **Mucin expression profiles in SAGE analysis.**
(DOCX)
