# Supplementary figures and images for: A GNAS Mutation Found in Pancreatic Intraductal Papillary Mucinous Neoplasms Induces Drastic Alterations of Gene Expression Profiles with Upregulation of Mucin Genes
Source: PLoS One. 2014 Feb 3;9(2):e87875. doi: 10.1371/journal.pone.0087875 (PMC3912139; doi:10.1371/journal.pone.0087875)

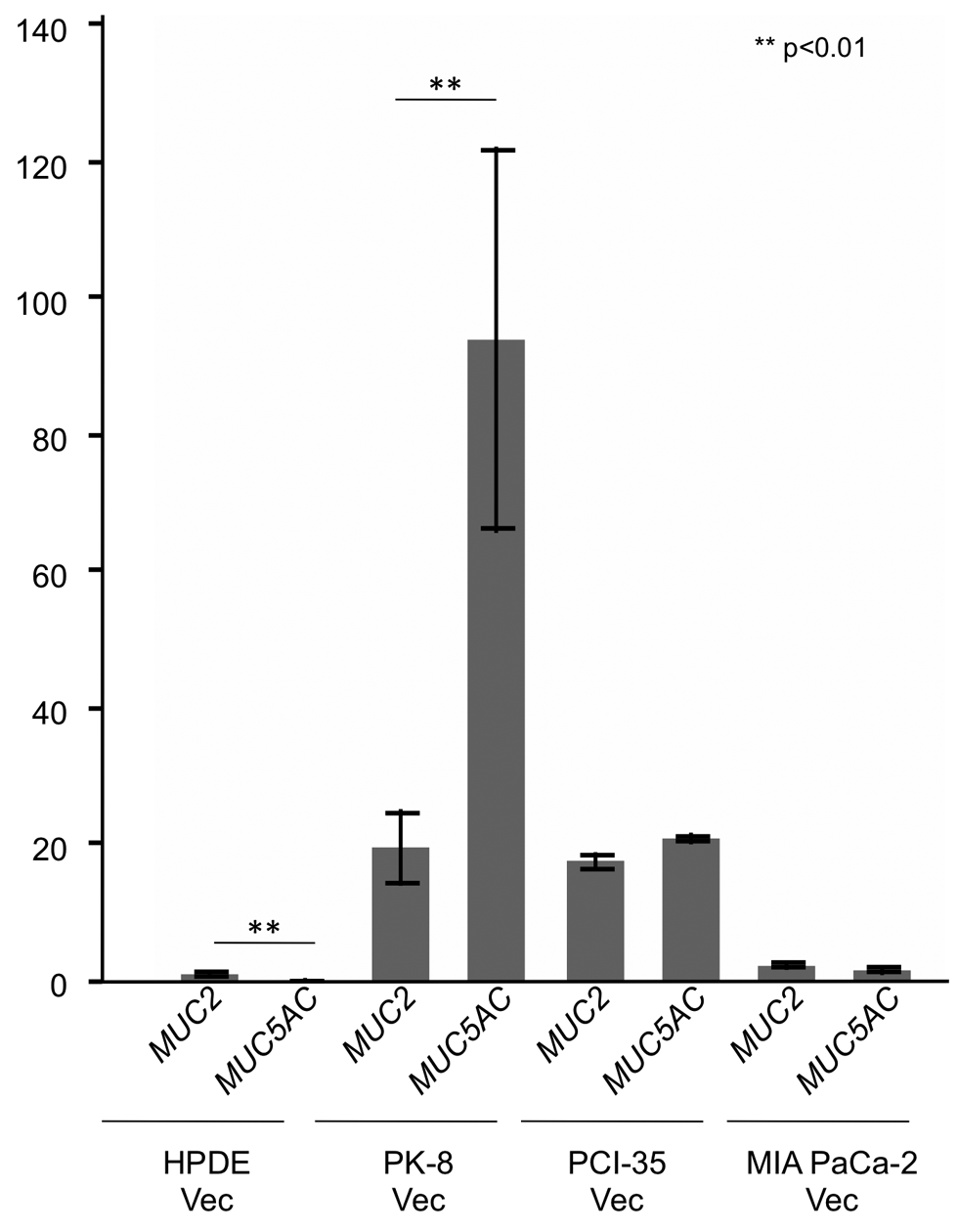

Supplement: Figure S1 — MUC2 and MUC5AC expressions among vector-transfectants. MUC2 and MUC5AC gene expression were evaluated by the quantitative real-time PCR method. Absolute values of each gene expression were converted into relative values to a value of MUC2 expression of HPDE vector-transfectant. Values of independently duplicated experiments were plotted with the range of one standard error and statistically compared. Two asterisks indicate p<0.01. (TIF) [file pone.0087875.s001.tif]

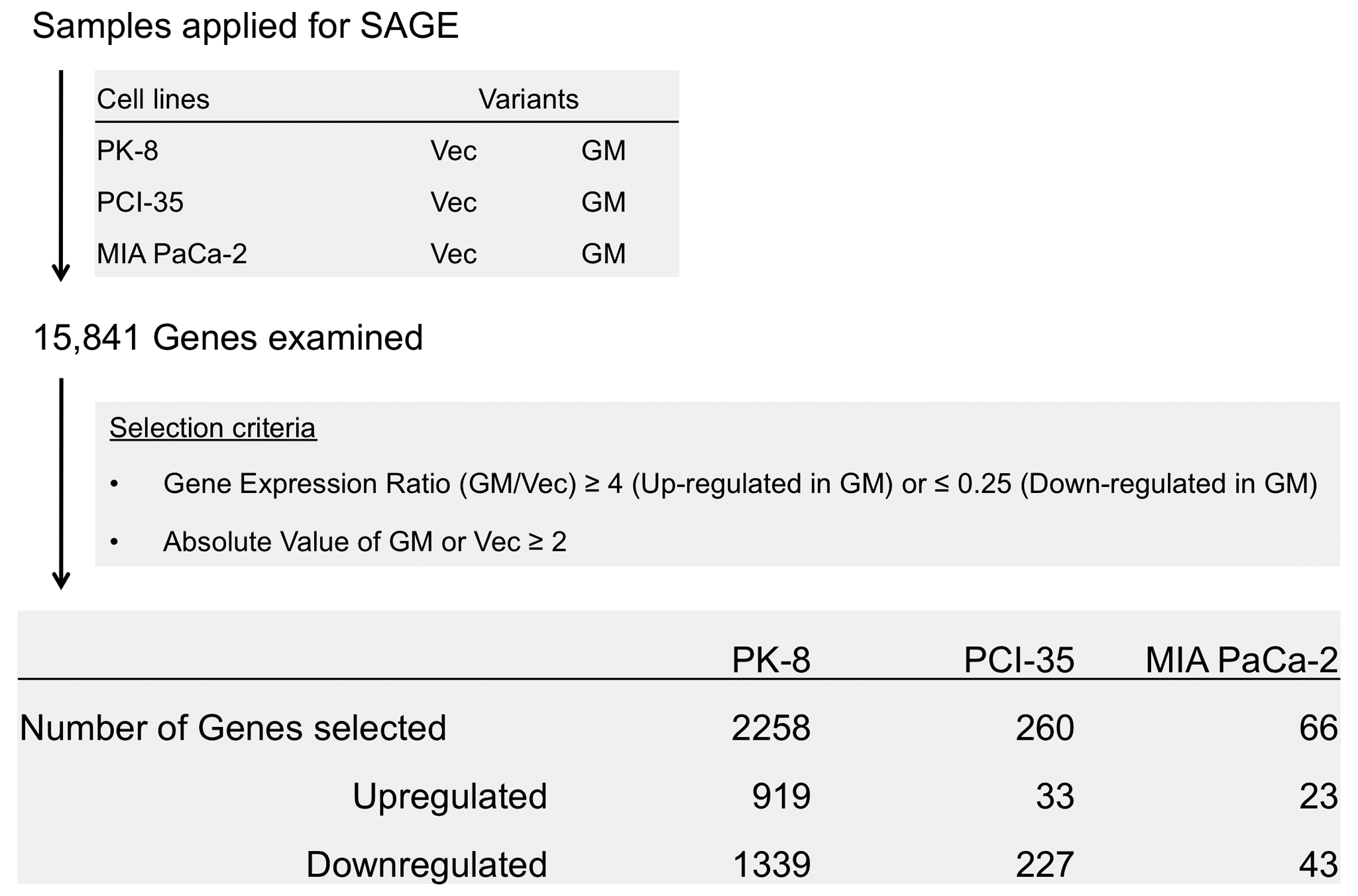

Supplement: Figure S2 — An algorithm for data processing in SAGE analysis. (TIF) [file pone.0087875.s002.tif]

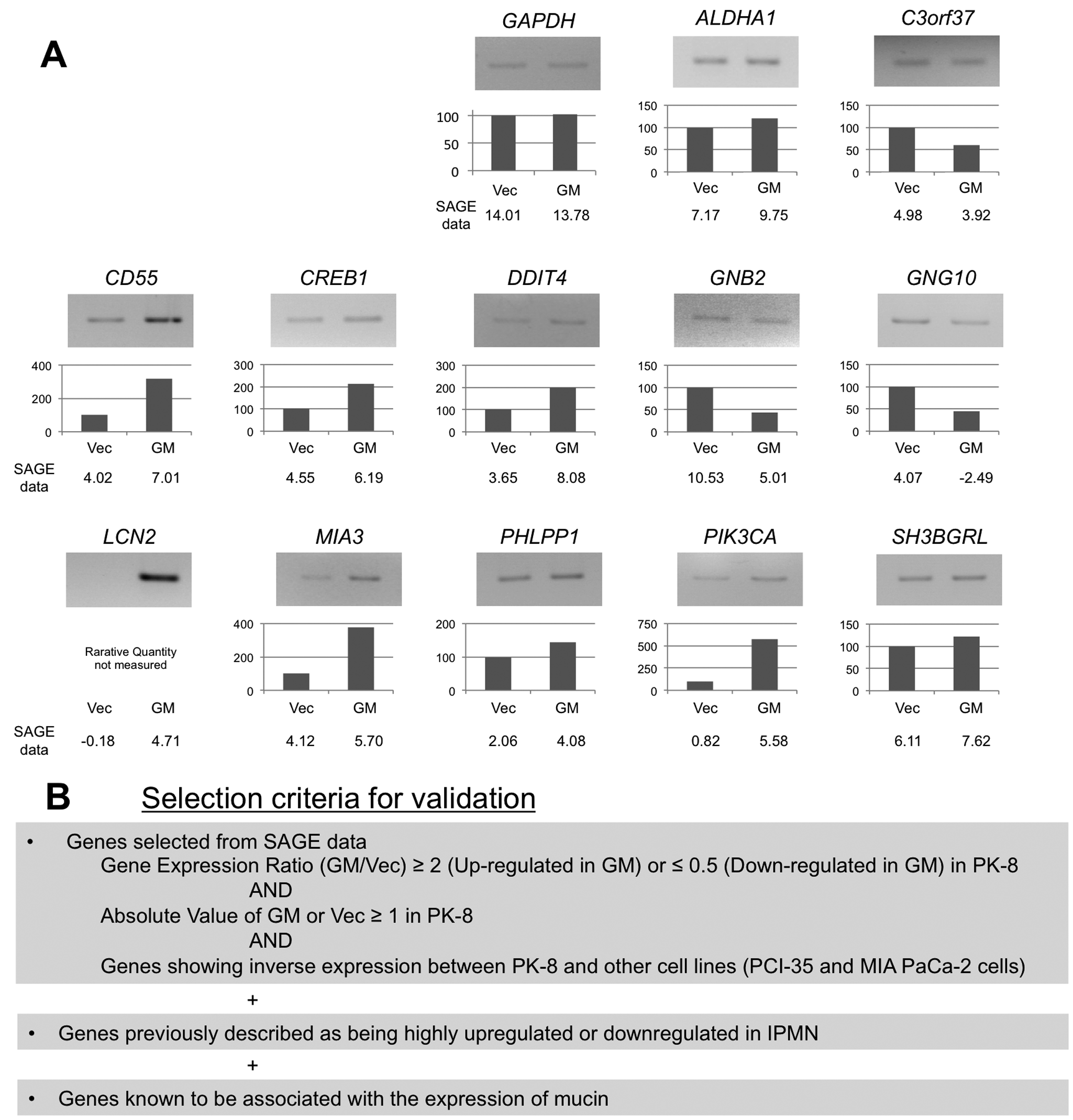

Supplement: Figure S3 — (A) Validation of SAGE data by semiquantitative PCR assay. Total RNA obtained from PK-8 transfected with vector (Vec) or the mutated GNAS (G201H) (GM), which was used in SAGE analysis, was reverse-transcribed and used for PCR with primers listed in Table S1. The optimized cycling conditions were determined for each gene, and the expression of GAPDH was represented as an internal control. Intensities of bands were digitally measured and illustrated with bar graphs below the images. Intensity of GM was demonstrated as relative value to Vec (%). Gene expression values in the SAGE analysis were shown at the bottom. (B) Selection criteria for validation. Specially considering the genes associated with mucin gene expressions, genes showing inverse expression between PK-8 and other cell lines (PCI-35 and MIA PaCa-2 cells) were nominated, because they demonstrated completely converse MUC2 and MUC5AC reactions to exogenously expressed GNAS in real-time qPCR experiments. Genes previously described as being highly upregulated or downregulated in IPMN and genes known to be associated with the expression of mucin were also validated. (TIF) [file pone.0087875.s003.tif]

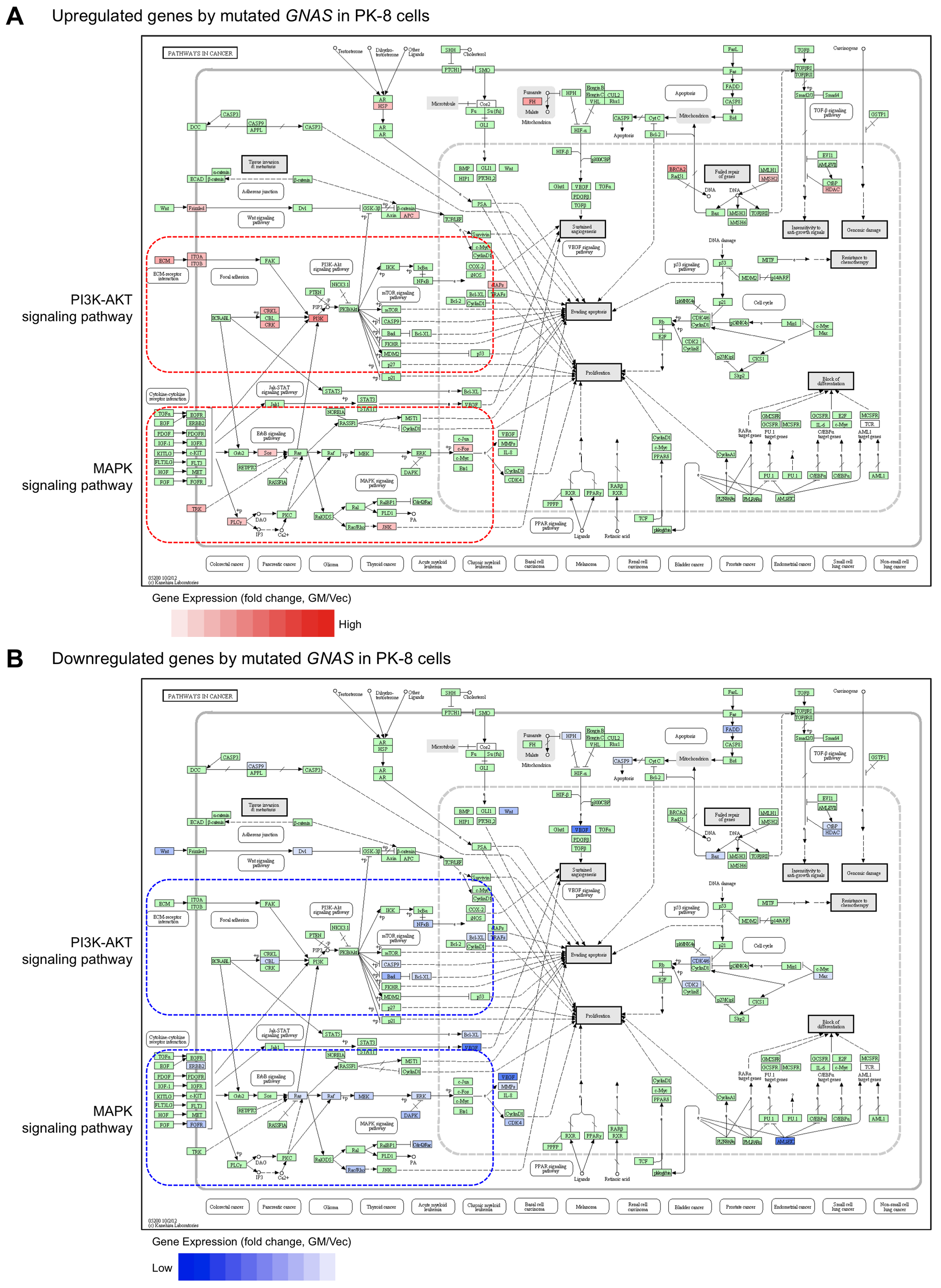

Supplement: Figure S4 — Alterations of gene expressions in signaling pathways. Genes of altered expressions in the ratio of the mutated GNAS transfectants to vector transfectants (GM/Vec) ≥4 or ≤0.25 in PK-8 in SAGE data were mapped on “Pathways in Cancer” in “Pathway Mapping” obtained from KEGG (http://www.genome.jp/kegg/). Panel A indicates upregulated genes while panel B indicates downregulated genes. (TIF) [file pone.0087875.s004.tif]

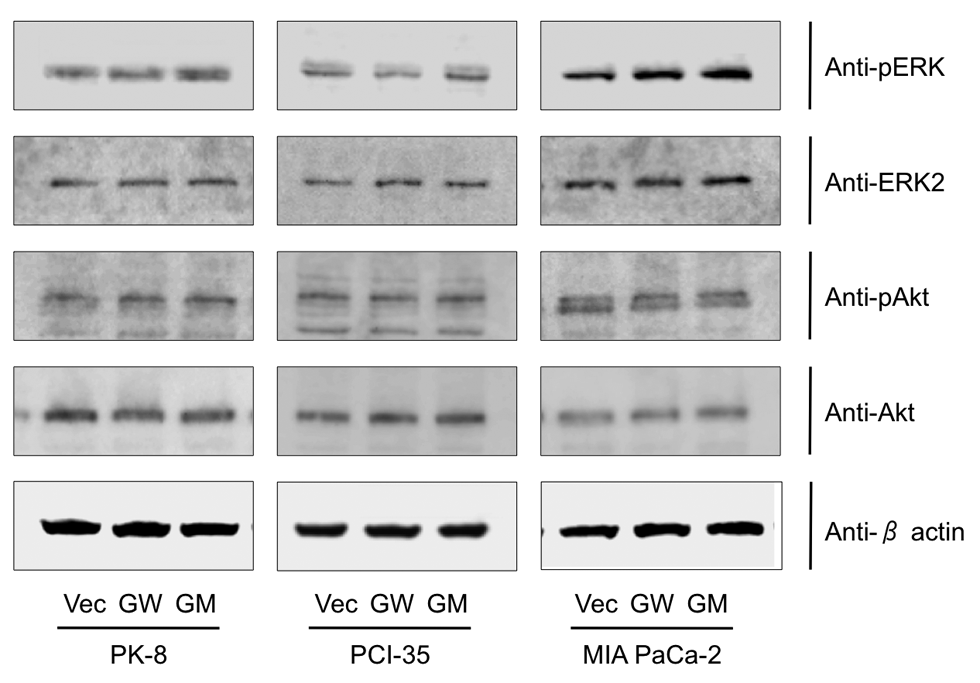

Supplement: Figure S5 — Immunoblots of total lysates of cells transfected with the vector (Vec), the wild-type GNAS -V5 (GW), and the mutated GNAS -V5 (R201H) (GM) probed with antibodies indicated in the right column. (TIF) [file pone.0087875.s005.tif]
