# Supplementary material for: A GNAS Mutation Found in Pancreatic Intraductal Papillary Mucinous Neoplasms Induces Drastic Alterations of Gene Expression Profiles with Upregulation of Mucin Genes
Source: PLoS One. 2014 Feb 3;9(2):e87875. doi: 10.1371/journal.pone.0087875 (PMC3912139; doi:10.1371/journal.pone.0087875)
Supplement: Table S1 — Primers used for RT-PCR reactions. (DOCX) [file pone.0087875.s006.docx]

**Table S1. Primers used for RT-PCR reactions**

| Gene | Forward primers (5'-3') | Reverse primers (5'-3') |
| --- | --- | --- |
| *GAPDH* | AGGTGGTGAAGCAGGCGTCG | ACCCTGTTGCTGTAGCCAAATTCG |
| *ALDH1A1* | GGGTGAATTGCTATGGCGTG | TGAAGAGCTTCTCTCCACTCTTG |
| *C30orf37* | CTCCTGTCGACTTGGTGGTC | GGACCACTGGGGAACATCTG |
| *CD55* | CACCACCTGAATGCAGAGG | CTAGCGTCCCAAGCAAACC |
| *CREB1* | CCCCAGCACTTCCTACACAG | CTTTCAGGTTGTGGCCAAGC |
| *DDIT4* | GTTCATCAGCAAACGCCCTG | GACACCCCATCCAGGTAAGC |
| *GNB2* | ATCCGACATCAATGCAGTGGC | GCCATGCCATCGTCGGTGAC |
| *GNG10* | CTGGCGTGGAGAGGATCAAG | GCTATAGGACCAGGCCACAG |
| *LCN2* | AACCAAGGAGCTGACTTCGG | AGCTCCCTCAATGGTGTTCG |
| *MIA3* | CCCTGTACCACCACCCATTC | GTGGTGGTGGGTATTCCTGG |
| *PHLPP1* | AAGCATGACCCTGTGGATCC | CACACTGGGATGGAGGAAGG |
| *PIK3CA* | AAAGGAGCCCAAGAATGCAC | GTTGTCCAGCCACCATGATG |
| *SH3BGRL* | GCAGCCAATGAAGAGAATCGG | AGGTTCATGCTTGCTGCTTTG |
