# Supplementary material for: A GNAS Mutation Found in Pancreatic Intraductal Papillary Mucinous Neoplasms Induces Drastic Alterations of Gene Expression Profiles with Upregulation of Mucin Genes
Source: PLoS One. 2014 Feb 3;9(2):e87875. doi: 10.1371/journal.pone.0087875 (PMC3912139; doi:10.1371/journal.pone.0087875)
Supplement: Table S2 — Mucin expression profiles in SAGE analysis. (DOCX) [file pone.0087875.s007.docx]

**Table S2. Mucin expression profiles in SAGE analysis**

|  | PK-8 | | | PCI-35 | | | MIA PaCa-2 | | |
| --- | --- | --- | --- | --- | --- | --- | --- | --- | --- |
| Gene | Vec | GM | Change (GM/Vec) | Vec | GM | Change (GM/Vec) | Vec | GM | Change (GM/Vec) |
| *MUC1* | 4.35 | 3.30 | Down | 7.25 | 8.18 | Up | 3.54 | 4.58 | Up |
| *MUC2* | NA | -2.49 | Up | NA | NA | - | NA | NA | - |
| *MUC5B* | NA | -1.49 | Up | -1.72 | -3.37 | Down | NA | NA | - |
| *MUC6* | NA | -2.49 | Up | NA | NA | - | -3.01 | NA | Down |
| *MUC15* | 1.66 | 2.91 | Up | 2.34 | 2.56 | Up | 1.90 | 1.72 | Down |
| *MUC16* | -1.40 | NA | Down | 1.87 | 2.33 | Up | NA | NA | - |
| *MUC20* | 3.12 | 3.22 | Up | 2.28 | 1.63 | Down | 1.57 | 0.67 | Down |
| *MUCL 1* | 1.02 | 0.32 | Down | NA | NA | - | NA | NA | - |

Values in this table were expressed in binary logarithm values of reads per mil1ion (log_2_ RPM). Down, downegulated in GM compared to Vec; GM, cells transfected with mutated *GNAS*; NA, not available; Up, upregulated in GM compared to Vec; Vec, cel1s transfected with an empty vector.
